# Effects of a parenting and unconditional cash transfer intervention on child development and maternal well-being in urban Bangladesh: medium-term follow-up of a cluster randomised controlled trial

**DOI:** 10.7189/jogh.16.04321

**Published:** 2026-11-06

**Authors:** Sheikh Jamal Hossain, Syed Moshfiqur Rahman, Sabbir Hossain, Sharmin Shiraji, Anisur Rahman, Helen Baker-Henningham, Jena Derakhshani Hamadani

**Affiliations:** 1 Maternal and Child Health Division, International Centre for Diarrhoeal Disease Research Bangladesh, Dhaka, Bangladesh; 2 Global Health and Migration Unit, Department of Women’s and Children’s Health, Uppsala University, Uppsala, Sweden; 3 School of Psychology, Bangor University, Bangor, UK

**Keywords:** child development, home stimulation, maternal depression, parenting, unconditional cash

## Abstract

**Background:**

Despite extensive evidence that early childhood parenting interventions improve children’s development, whether these effects are sustained in the longer term remains uncertain. We measured the medium-term effects of a parenting and cash transfer programme compared to a cash transfer alone on child, parenting, and maternal outcomes.

**Methods:**

In the original trial, we recruited twenty clusters in Rangpur City Corporation of Bangladesh and randomised them equally to intervention (parenting and unconditional cash, 299 participants) or control groups (unconditional cash transfer only, 300 participants). Participants were mothers of children aged 6–16 months on enrolment. Here, we followed up mother/child dyads five years after the intervention when children were aged 6.2 to 8.4 years. The parenting intervention involved fortnightly home visits by trained facilitators for one year. Funds for the cash transfer were provided by the Government of Bangladesh. The primary child outcome was IQ, while secondary ones included reading and mathematics skills. Parenting outcomes included quality of home stimulation and mothers’ childcare knowledge. Maternal outcomes included depressive symptoms, empowerment, violence exposure and quality of life.

**Results:**

Out of the 599 participants enrolled in the initial trial, 555 (92.7%) mother/child dyads participated in this five-year follow-up. We observed no benefits of the combined intervention compared with cash alone for the primary outcome of child IQ (effect size = 0.11; 95% confidence interval = −0.04, 0.27); for the secondary outcomes, we observed benefits for reading, but not for mathematics skills. We also found benefits for home stimulation, mothers’ childcare knowledge, depressive symptoms, and empowerment, but none for mothers’ violence exposure and quality of life.

**Conclusions:**

Although we found no benefit for the primary outcome of child IQ, a combined parenting and cash transfer intervention showed medium-term benefits compared to cash transfer alone for children’s reading skills, home stimulation, mothers’ childcare knowledge, depressive symptoms, and empowerment. However, as these findings are related to secondary outcomes of interest for our study, they should be considered as hypothesis-generating only.

**Registration:**

clinicaltrials.gov: NCT03753646 (original cluster randomised controlled trial), NCT07432971 (follow-up)

Experiences in early childhood affect brain architecture and development, and can impact children’s education, income, and quality of life in the long term [1]. Systematic reviews and meta-analyses have reported that early parenting interventions focused on early childhood development alone and combined with health, nutrition or cash transfer programmes benefit children’s development immediately after intervention in low- and middle-income countries [2–4]. In the context of Bangladesh, several randomised controlled trials of parenting interventions integrated into health, nutrition, or social protection programmes, including unconditional cash transfer, have shown benefits to child development immediately after the intervention [5–11].

Unconditional cash transfer alone has been shown to benefit parenting behaviour [12], as well as improve child birth registration, vaccination uptake, and school attendance [13], child health [14], and development outcomes [15]. However, in a small study conducted in rural Bangladesh, unconditional cash transfer did not lead to improvements in child development [8]. Some research has shown that combining an early parenting intervention focused on early childhood development with cash transfer can help improve disadvantaged children’s health and development [8,16–19]. Yet in contrast, a systematic review and meta-analysis showed that cash transfer combined with parenting programmes was not more effective than cash transfers alone in promoting children’s overall cognitive development, although there was substantial heterogeneity in the few studies included in the analysis [20]. Besides improvements in child development, there is some evidence that parenting interventions can lead to concurrent benefits to maternal outcomes, including well-being and empowerment, although these effects were inconsistent across studies [21]. Whether unconditional cash transfers alone can improve maternal well-being is also unclear [22–24].

Despite evidence of the short-term benefits of parenting interventions, a systematic review and meta-analysis of trials conducted in low- and middle-income countries reported that their medium-term (age 6–12 years) and longer-term (age >12 years) effects on children’s development are uncertain [3]. One study conducted in Jamaica repeatedly followed participants across the life course and reported benefits to child IQ at age 11–12 years [25], as well as IQ, school achievement, and mental health at the age of 17–18 years and 22 years [26,27]; to a reduction in violent behaviour at the age of 22 years [27]; and increased earnings at the age of 31 years [28]. However, no benefits were found for child IQ, language, and school performance at the age of 7–8 years [29]. Other follow-up studies of early childhood parenting interventions have shown mixed results. In Bangladesh, a study which followed up participants in the medium term, *i.e.* to six years after the parenting intervention, found sustained effects on children’s IQ at the age of 8–9 years for children without anaemia, but not for children with iron-deficiency anaemia [30]. A follow-up of a trial in Colombia evaluating the effect of a combined cash transfer and an early childhood parenting intervention found no benefits to children’s development at two years after the intervention, when the children were 4.5–5.5 years of age [31].

The theoretical rationale for evaluating the medium-term effects of early childhood parenting interventions is that early childhood is characterised by rapid brain development and heightened neuroplasticity, making it a sensitive period during which a responsive and stimulating caregiving environment may have lasting effects on children’s development [32]. However, the quality of the environment children grow up in, both at home and at school, may either reinforce or attenuate these early gains [33]. The fade-out and re-emergence hypothesis proposes that early benefits may not be fully apparent in middle childhood IQ, but that they may re-emerge as more complex cognitive and social skills develop in adolescence and adulthood [33].

During 2018–2020, we conducted a cluster randomised controlled trial in an urban area of Bangladesh that compared unconditional cash transfer alone to an intervention combining an early childhood parenting intervention with an unconditional cash transfer programme implemented by the Government of Bangladesh (GoB). We found that, after one year of the intervention, the combined parenting and cash transfer intervention led to significant and meaningful improvements to children’s cognition (effect size (ES) = 0.42), language (ES = 0.38), and motor development (ES = 0.17) compared to cash transfer alone [5]. We also observed benefits for the quality of home stimulation, as well as mothers’ knowledge of childcare, exposure to domestic violence, quality of life, and depressive symptoms [5,34]. Here, we report on the findings of our medium-term follow-up five years after the end of the intervention, through which we wanted to assess whether the benefits of the combined parenting and cash transfer intervention for child, parenting, and maternal outcomes were sustained.

## METHODS

### Original trial

The original trial was a cluster randomised controlled trial delivered in 20 of the 33 wards (geographically defined administrative government units of a city corporation) in Rangpur City Corporation, Bangladesh [5]. The wards were randomly selected and allocated in a one-to-one ratio to the intervention group (parenting plus unconditional cash transfer) or the control group (unconditional cash transfer only), with randomisation done by ward to reduce contamination across clusters. The allocation was conducted by an independent statistician who was not involved in the trial.

The GoB’s unconditional cash transfer was delivered through in a social protection programme that enrolled urban working mothers with a monthly income BDT <1500 (USD <17.6, per the World Bank 2020 conversion rates), fewer than three children, and permanent residence in an urban area [35,36]. The original trial included mothers who were participating in the GoB’s programme and had a child aged 6–16 months. Thirty mother/child dyads were enrolled in each ward, giving a total sample size of 599 mother/child dyads: 299 in the parenting plus unconditional cash transfer group and 300 in the unconditional cash transfer only group (**Figure 1**). The enrolment period lasted from 20 December 2018 to 1 March 2019 [5].

**Figure 1:**
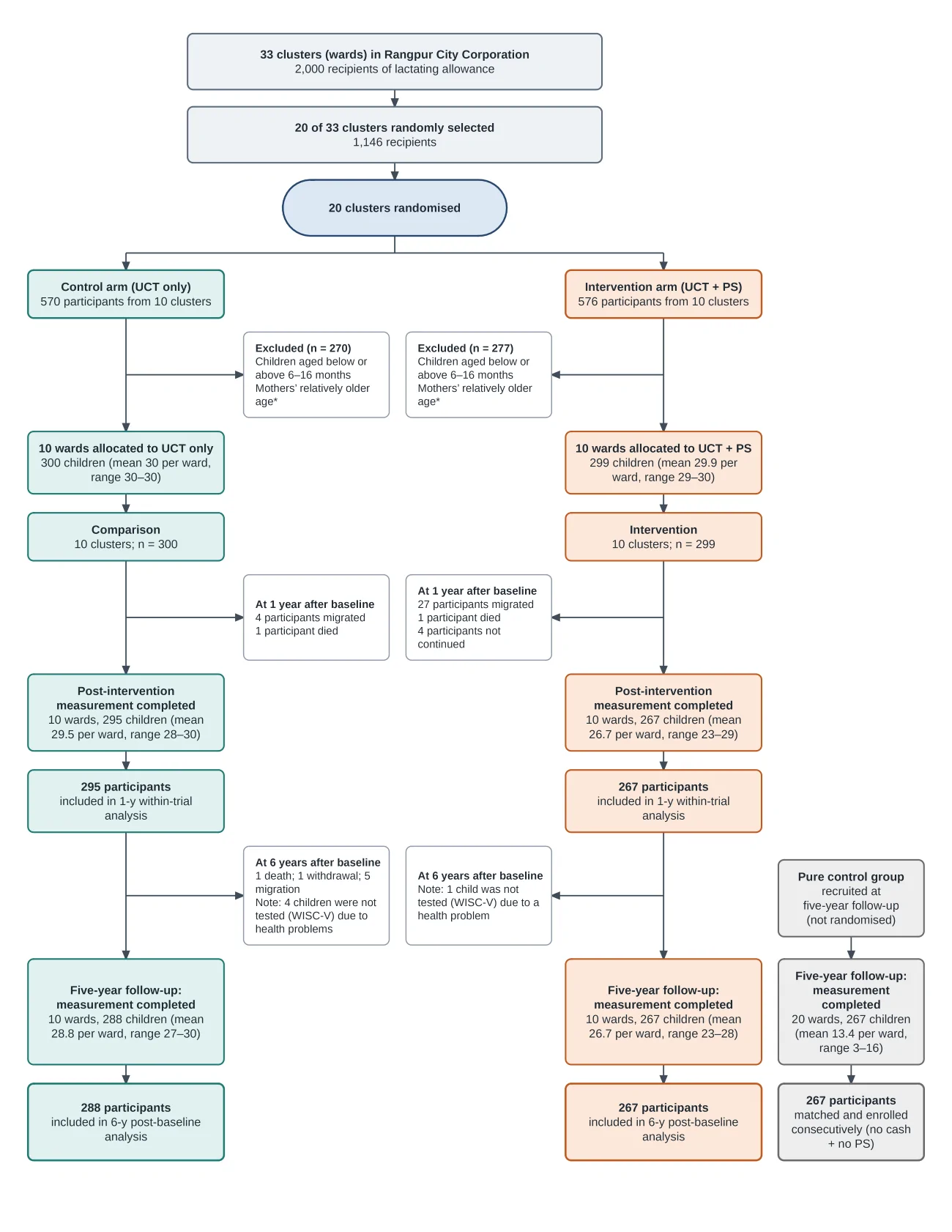
Trial profile. PS – psychosocial stimulation (parenting intervention), UCT – unconditional cash transfer, WISC-V – Wechsler Intelligence Scale for Children, Fifth Edition. *In clusters with more than 30 eligible mothers on the list provided by the Ministry of Women and Children Affairs, Government of Bangladesh, the 30 youngest mothers were enrolled.

### Study design and participants at five-year follow-up

We conducted this follow-up study from December 2024 to March 2025, when the children were 6.2–8.4 years of age. All participants from the original trial were considered eligible and were contacted using previously collected mobile phone number and locator information.

At this follow-up, we added a pure control arm that included participants who had not received parenting support or participated in the GoB social protection programme. We selected this sample by matching for the children’s age (with an acceptable divergence of six months above or below the mean age of children already included in the study), children’s sex, and family income and location, whereby field staff visited the study area and conducted a door-to-door survey to identify eligible children. The target was fifteen mother/child dyads per cluster, enrolled in the order they were identified. Because of time constraints, we were unable to reach this target in several clusters (range 3–16 per cluster), giving a sample of 267 mother/child dyads (**Figure 1**).

### Intervention

Full details on the intervention are described elsewhere [5,34]. In short, through the GoB’s unconditional cash transfer programme, mothers in both the intervention and comparison groups received BDT 800 (USD 9.4, per the World Bank 2020 conversion rates) per month for three years from pregnancy onwards, with the funds delivered directly through their mobile phone (bKash account, a money transfer service) or to their banking account. The mothers in the intervention group also participated in one year of parenting support delivered by a female home visitor resident in the city corporation and hired through the research project. The intervention team included six home visitors aged 20–28 years, five of whom had undergraduate degrees and one a higher secondary degree. The home visitors were trained on the adapted Reach Up parenting intervention for 10 days and received weekly field supervision throughout the implementation of the intervention [37]. They conducted fortnightly home visits lasting for approximately one hour, during which they demonstrated play techniques and encouraged the mothers to practise the activities with their child during the visit and to continue them between visits. Low-cost toys made from commonly available recyclable materials and picture books were left in the home and exchanged after each visit.

### Outcomes measured at five-year follow-up

We considered child, parenting, and maternal outcomes in our analysis (**Table 1**). The primary outcome was child IQ measured using the Wechsler Intelligence Scale for Children, 5th edition, an internationally standardised tool for global developmental assessment of children aged 6–16 years [39]. Its previous iteration has been used in other studies in Bangladesh, where it showed good internal consistency and test–retest reliability [40,41]. We also measured children’s achievement in reading and numeracy using the Early Grade Reading Assessment [42] and Early Grade Mathematics Assessment [43], respectively.

**Table 1:** Description of outcome measures at five-year post-intervention follow-up

Child outcomes	
FSIQ	Assessed using the WISC-V and reflects the child’s overall intellectual ability.
EGRA	The EGRA assessment has four components: listening comprehension, letter recognition, random non-word, and oral reading and comprehension. We used listening comprehension and letter recognition only and the scores for these components were summed to form the total score. We dropped random non-word and oral reading and comprehension, as most children scored 0 on these components.
EGMA	The EGMA assessment tool has five components: number identification, number discrimination, missing number identification, addition and subtraction, and word problems. The sum of all five components gives the final EGMA score.
**Parenting outcomes**	
Child stimulation environment at home	Assessed using HOME – Middle Childhood tool. The tool has 8 components (responsivity, maturity, emotional tone, opportunities, enrichment, companionship, integration, and environment), which are summed up to obtain a total home score (range = 0 to 43).
Maternal knowledge on childcare	Measured through an interviewer-administered questionnaire and included questions on childcare knowledge and practice. A total parenting knowledge score (range = 0–60) is computed by summing the individual scores of all items.
**Maternal outcomes**	
Depression	Measured using SRQ-20 questionnaire. Final values range from 0 to 20, with higher scores indicating higher depressive symptoms.
Empowerment	Measured by a validated tool used in the Bangladesh DHS [38]. We used two components of maternal empowerment: maternal freedom of mobility and decision sharing for financial expenditure, with additional questions added related to childcare and development. We calculated the total empowerment score (range = 10–30) by summing the scores for all individual components, with higher values indicating greater empowerment.
Quality of life	Measured by using WHOQOL-BREF, which comprises 26 items spanning 4 domains: physical health, psychological health, social relationship, and environment. Each domain score is converted from a raw score to a transformed score, with final values for each domain ranging from 0 to 100, where higher scores indicate a higher quality of life. Each domain was considered as an outcome.
Exposure to violence	A binary outcome indicating whether the mother experienced any form of violence (physical, mental, or sexual) in the past month. Coded as 1 if mother was exposed to violence and 0 otherwise.

DHS – Demographic and Health Survey, EGMA – Early Grade Mathematics Assessment, EGRA – Early Grade Reading Assessment, FSIQ – full-scale IQ, HOME – Home Observation for Measurement of the Environment, SRQ-20 – Self-Reporting Questionnaire-20, WHOQOL-BREF – World Health Organization Quality of Life – Brief Version, WISC-V – Wechsler Intelligence Scale for Children – Fifth Edition

For parenting outcomes, we measured the quality of stimulation in the home using the middle childhood version of Home Observation for Measurement of the Environment [44], and maternal knowledge on childcare using a questionnaire developed for the original trial and administered at baseline [5].

For maternal outcomes, we measured depressive symptoms through the Self-Reporting Questionnaire – 20 [45] and quality of life using the World Health Organization Quality of Life – Brief Version [46]. We assessed women’s empowerment using a questionnaire developed previously for Bangladesh following the women empowerment measurement framework for married women [38,47,48]. This questionnaire included two components of maternal empowerment: maternal mobility and decision sharing for household financial expenditure. We further added questions on decision-making related to child development, such as mothers’ ability to purchase toys or picture books for their children, and to take their child to a health facility when ill (**Table 1**). We measured violence against women using a World Health Organization multi-country questionnaire on violence against women [49].

We also collected the demographic and socioeconomic characteristics of the participants and their families, including the duration of breastfeeding, parental education, and family wealth. The weight and height of the children were measured using standardised techniques.

The Wechsler Intelligence Scale for Children, 5th edition, had an internal consistency (Cronbach’s alpha) of 0.75 in this sample and a two-week test–retest intraclass correlation coefficient (ICC) of 0.80. Test–retest ICCs for the Early Grade Reading Assessment and the Early Grade Mathematics Assessment were 0.83 and 0.81, respectively. The other measures had a mean Cronbach’s alpha of 0.76 (range 0.56–0.87).

### Procedure for data collection at five-year follow-up

Two groups of data collectors comprising six interviewers and five child testers collected data over two sessions with each family. First, the interviewers visited the homes and interviewed mothers to collect data on socioeconomic characteristics, knowledge of childcare, women’s empowerment (freedom of mobility and decision sharing on household purchasing ability of the mothers), and the quality of the home environment. Mothers were then invited to come to a centre (a school/community centre/local club/residence of a local popular person) near their residence with their child, where the testers assessed children’s IQ and school achievement, mothers’ depressive symptoms, quality of life, and violence against the mothers. The testers also measured children’s height and weight.

All data collectors and testers had at least an undergraduate or master’s degree. Child testers were trained by a master trainer with extensive experience in administering the pertinent child assessments, and each tested at least 10 children who were not part of the study before initiating data collection. Interviewers were trained by another trainer with experience in the middle-childhood Home Observation for Measurement of the Environment and parent questionnaires. All data collectors and testers were masked to the participants’ group allocation (intervention or comparison). However, it is possible that some mothers may have disclosed that they had received home visits during the child’s early childhood. All data collectors and testers worked together within the same cluster, completing data collection in one cluster before moving together to the next. Interobserver reliability between the trainers and assessors was >90% during training and ongoing data collection for all measures.

### Statistical analysis

In a parallel trial, 165 participants per group would be required to detect an effect size of 0.31 standard deviation (SD) for the primary outcome of children’s cognitive development, assuming 80% power with a 95% confidence level. With a cluster size of 30 (*i.e.* 30 mother/child dyads per cluster) and an intra-cluster correlation coefficient of 0.02 (based on the post-test data), the design effect is 1.58, giving a required sample size of 261 per group. At this five-year follow-up, we assessed 550 children (266 intervention and 284 control).

We used multilevel regression analyses to assess the effect of intervention on child, parenting, and maternal outcomes, with linear mixed-effects models applied for continuous outcomes and generalised linear mixed-effects models with a logit link for the binary outcome of violence against mothers. Fixed effects in the models included a constant term, the respective baseline outcome, the intervention group, testers/interviewer, and baseline covariates which were statistically significantly different between the groups or that were different between those lost and assessed at this follow-up at a *P*-value <0.1. Wards (clusters) were entered as a random effect. The normality assumption of the residuals was also checked.

We similarly used multilevel regression analyses to evaluate the difference between the cash transfer only *vs*. pure control group, where the fixed effects included a constant term, the intervention group, testers or interviewer, and covariates which were statistically significantly different between the groups at a *P*-value <0.1. These comparisons were non-randomised and exploratory.

We calculated effect sizes for continuous outcomes by dividing the adjusted regression coefficient by the SD of the control group for each dependent variable. As they were exploratory, the analyses for the secondary outcomes were not adjusted for multiple comparisons. All analyses were conducted with *R*, version 4.3.1 (R Core Team, Vienna, Austria).

## RESULTS

The original trial included 599 participants, of whom 562 (93.8%) completed the study. At this five-year follow-up, 555 of the original sample (92.7% follow-up; 89% intervention, 96% control) were retained and only one participant did not give consent to participate. Three children died from baseline to follow-up (**Figure 1**). We could not test five children (four in the control and one in the intervention group) due to health problems, but could interview their mothers. There were no significant differences between those tested and those lost to follow-up (Table S1 in the **Online Supplementary Document**).

The children’s mean age at follow-up was 6.9 years (SD = 0.3) and 50.3% were female. One fifth of the mothers (20.9%) had less than five years of education. The intervention and control groups were reasonably well balanced at enrolment, except for significant differences in terms of violence against mothers, household wealth index, and children’s language scores (**Table 2**). We controlled for these variables in all analyses.

**Table 2:** Characteristics at enrolment of participants followed up at five years by study group*

	UCT (n = 288)	UCT with parenting (n = 267)	*P*-value
**Background characteristics**			
Children’s age in months	11.2 (3.2)	11.4 (3.1)	0.60
Children’s sex, girls, n (%)	153 (52.9)	127 (47.6)	0.20
Mothers’ BMI	23.0 (4.5)	22.8 (3.9)	0.50
Mothers’ age in years	25.7 (5.4)	25.7 (4.7)	0.90
Mothers’ education in years	6.9 (3.4)	7.2 (3.6)	0.30
Fathers’ education in years	5.5 (4.2)	5.6 (4.4)	0.70
Wealth Index, n (%)			<0.001
*Poor*	118 (41.2)	68 (25.5)	
*Middle*	88 (30.4)	95 (35.6)	
*Rich*	82 (28.4)	104 (39.0)	
**Child outcomes**			
Cognitive development	93.3 (10.5)	94.7 (11.2)	0.13
Language development	90.4 (12.1)	92.5 (13.0)	<0.05
Motor development	93.7 (14.3)	93.8 (15.2)	0.90
Length-for-age z-score	−1.1 (1.1)	−1.1 (1.2)	0.70
Weight-for-age z-score	−0.7 (1.2)	−0.6 (1.0)	0.11
Weight-for-length z-score	−0.2 (1.3)	0.0 (1.1)	0.06
**Parenting outcomes**			
Child stimulation environment (FCI scores)	5.4 (3.3)	5.9 (3.3)	0.10
Mothers’ knowledge on parenting	31.3 (10.6)	31.7 (11.0)	0.71
**Maternal outcomes**			
Depressive symptoms	8.4 (4.7)	9 (4.9)	0.20
Exposure to violence, n (%)	126 (43.6)	140 (52.4)	0.04
Quality of life			
*Physical*	65.8 (13.7)	64.8 (14.4)	0.40
*Psychological*	60.4 (13.1)	60.8 (12.0)	0.70
*Social relationship*	57.9 (15.0)	58.5 (14.7)	0.60
*Environmental*	49.0 (11.8)	50.7 (10.9)	0.08

BMI – body mass index, FCI – family care indicators, UCT – unconditional cash transfer

*Data are presented as mean (standard deviation) unless specified otherwise.

There was no effect of the combined parenting and unconditional cash transfer intervention at five-year follow-up on the primary outcome of children’s FSIQ scores (adjusted effect size (aES) = 0.11; 95% CI = −0.04, 0.27) compared to the group that received cash transfer alone (**Table 3**). There was also no benefit to children’s mathematics skills (aES = 0.11; 95% CI = −0.05, 0.28), but we did observe positive effects of the combined intervention on children’s reading skills (aES = 0.22; 95% CI = 0.03, 0.41). We also observed its benefits for the quality of the home environment (aES = 0.55; 95% CI = 0.25, 0.85), mothers’ knowledge of childcare (aES = 0.96; 95% CI = 0.64, 1.29), maternal depressive symptoms (aES = −0.28; 95% CI = −0.44, −0.11), and women’s empowerment (aES = 0.35; 95% CI = 0.01, 0.70). There were no benefits of the combined intervention to mothers’ quality of life and exposure to violence.

**Table 3:** Effects of parenting on outcomes (parenting + cash *vs*. cash only) at five-year follow-up*

	*β* (95% CI)	Adjusted ES (95% CI)	ICC	*P*-value
**Child outcomes**				
FSIQ	1.26 (−0.49, 3.01)	0.11 (−0.04, 0.27)	0.00	0.16
EGRA	2.88 (0.40, 5.36)	0.22 (0.03, 0.41)	0.01	0.02
EGMA	2.38 (−1.11, 5.87)	0.11 (−0.05, 0.28)	0.01	0.18
**Parenting outcomes (n = 555)**				
Home total score	3.88 (1.75, 6.00)	0.55 (0.25, 0.85)	0.14	<0.001
Knowledge on childcare	9.34 (6.15, 12.54)	0.96 (0.64, 1.29)	0.16	<0.001
**Maternal outcomes**				
Total empowerment (n = 555)	1.58 (0.02, 3.13)	0.35 (0.01, 0.70)	0.13	0.047
Depression (n = 553)	−1.37 (−2.18, −0.56)	−0.28 (−0.44, −0.11)	0.01	0.001
Exposure to violence (n = 553), OR (95% CI)	0.82 (0.53, 1.26)		0.01	0.36
Quality of life (n = 553 unless indicated)				
*Physical*	1.32 (−0.99, 3.64)	0.10 (−0.07, 0.28)	0.01	0.26
*Psychological*	2.00 (−0.98, 4.99)	0.16 (−0.08, 0.39)	0.05	0.19
*Social (n = 520)*†	2.41 (−0.06, 4.87)	0.16 (−0.00, 0.33)	0.00	0.06
*Environmental*	0.97 (−1.19, 3.13)	0.08 (−0.09, 0.24)	0.01	0.38

CI – confidence interval, EGMA – Early Grade Mathematics Assessment, EGRA – Early Grade Reading Assessment, ES – effect size, FSIQ – full-scale IQ, ICC – intracluster correlation coefficient, OR – odds ratio, *β* – regression coefficient

*Analyses include 550 participants unless indicated otherwise. We used mixed-effects linear regression models for continuous variables and generalised linear mixed models with logit link for binary outcomes. All models were adjusted for baseline outcomes (if available), baseline characteristics that were significantly different between groups (children’s language development score, children’s weight-for-length z-score, mothers’ exposure to violence, mothers’ environmental quality of life, and wealth index), children’s age at follow-up, children’s sex, and tester/interviewer. In the model for FSIQ, the baseline outcome was baseline cognitive score, and the language score was not included, as the two were highly correlated. †The social relationship domain of the World Health Organization Quality of Life – Brief Version contained one question on sexual life that many mothers did not answer (16 in the intervention group and 17 in the UCT group).

In the non-randomised comparison of the pure control arm with the unconditional cash transfer arm, the pure control group scored significantly lower than the unconditional cash transfer group for mothers’ education, fathers’ education, and wealth index (Table S2 in the **Online Supplementary Document**). We controlled for these variables in all analyses.

We found no benefits of cash transfer alone for the primary outcome of child full-scale IQ. There were also no benefits for children’s achievement in reading and mathematics. Significant benefits of cash transfer alone were found for the quality of the home environment (aES = 0.78; 95% CI = 0.59, 0.97) and for maternal empowerment (aES = 0.66; 95% CI = 0.47, 0.85). There were no differences between the groups in mothers’ depressive symptoms or exposure to violence, and no consistent differences in quality of life (Table S3 in the **Online Supplementary Document**).

## DISCUSSION

In this study comparing the medium-term impact of a combined early childhood parenting plus cash transfer intervention with cash transfer alone, we found no medium-term benefits to the primary outcome of child IQ. Exploratory analyses showed benefits for several secondary outcomes, including children’s reading achievement, the quality of home environment, mothers’ knowledge of childcare, maternal depressive symptoms, and women’s empowerment. We identified no benefits for children’s mathematics achievement, mothers’ quality of life, and mothers’ exposure to violence.

The positive concurrent effects of the combined parenting plus cash transfer interventions on early childhood development in this population were not sustained for children’s IQ at age 6.9 (SD = 0.3) years. This is consistent with a systematic review and meta-analysis, which found that the benefits of early childhood interventions often attenuate or fade during the middle childhood period (four-to-nine years) [3], and with follow-up studies from Colombia [31] and Rwanda [50]. A longitudinal study in Jamaica also found no benefits to child IQ, language, or school performance in middle childhood [29], although the intervention led to significant benefits across multiple domains in adolescence and adulthood [26–28]. This pattern fits the fade-out and re-emergence hypothesis, which proposes that gains to foundational skills from early interventions may fade, while benefits to more complex skill acquisition emerge later [33].

Among the secondary outcomes, we observed the most robust effects of the intervention for home stimulation (aES = 0.55) and maternal knowledge of childcare (aES = 0.96), both showing moderate-to-large effects with narrow confidence intervals. Reading achievement showed a small but statistically significant effect (aES = 0.22). The finding regarding impacts on women’s empowerment (aES = 0.35; 95% CI = 0.01, 0.70) should be treated with particular caution: the confidence interval barely excludes zero and the effect is small-to-moderate. The reduction in depressive symptoms (aES = −0.28) appears more robust.

In the comparison between participants receiving cash transfer alone and the newly enrolled non-randomised pure control group, we found no benefits of cash transfer alone for children’s IQ, reading achievement, and mathematics achievement. However, cash transfer alone benefited home environment quality and mothers' empowerment, consistent with other evidence [31,51]. The amount and duration of unconditional cash transfers are also likely to affect programme effectiveness, although it is not clear what amount and duration are most effective [52]. In the GoB’s programme, the cash transfer sum is equivalent to approximately 10% of the families’ monthly income and is given for three years.

This study provides preliminary evidence that combining structured parenting support with an existing government cash transfer programme leads to sustained benefits for home stimulation, maternal knowledge of childcare, depressive symptoms, and child reading skills five years after intervention. If replicated in larger and more diverse samples, these findings could support the integration of parenting components into national social protection systems. For example, benefits to child reading skills and home stimulation are important, as they may shape long-term educational trajectories [53] and future earnings [54]. Reductions in maternal depressive symptoms and improvements in women’s empowerment may improve mothers’ caregiving capacity, consistency, emotional bonding, and household harmony [55,56]. Furthermore, utilising social protection systems as platforms for parenting interventions focused on early childhood development extends the reach of parenting support to millions of poor families, with potential for high social returns.

This study has several strengths: it was a five-year follow-up of a cluster-randomised controlled trial, had low attrition (7.3% loss to follow-up), assessors were masked to intervention status, the measures had good psychometric properties, and a comprehensive range of child, parenting, and maternal outcomes were measured. There were also some limitations. Although contamination between intervention and control wards is unlikely given the geographic separation, we acknowledge this cannot be entirely excluded. The sample was small (ten clusters per group) and was powered to detect an ES of 0.31 SD. As the observed IQ effect was an aES of 0.11 SD, the null primary finding should be interpreted with caution, as a small true effect cannot be excluded. We also conducted this study in a single urban area, which limits the generalisability of our findings. Participants in the non-randomised pure control arm, who did not participate in the GoB’s social protection programme during early childhood, had lower parental education and wealth than those in the cash transfer only arm, indicating that the most disadvantaged families failed to access the social protection programme. This finding is itself relevant to policy. Residual confounding in the non-randomised comparisons cannot be excluded despite statistical adjustment.

## CONCLUSIONS

Our findings suggest that early parenting interventions integrated into social protection systems may have sustained positive effects on parenting and maternal outcomes, with modest improvements in children’s early reading skills. Given that many of these outcomes were secondary and exploratory, the results should be treated as preliminary evidence.

## Additional material


Online Supplementary Document

## Data Availability

**Data availability:** The data can be obtained from the corresponding author upon request.
