# Supplementary material for: Effects of a parenting and unconditional cash transfer intervention on child development and maternal well-being in urban Bangladesh: medium-term follow-up of a cluster randomised controlled trial
Source: J Glob Health. 2026 Nov 6;16:04321. doi: 10.7189/jogh.16.04321 (PMC13741169; doi:10.7189/jogh.16.04321)
Supplement: Supplementary file 1 [file jogh-16-04321-s001.pdf]

Supplement to: Hossain SJ, Rahman SM, Hossain S, Shiraji S, Rahman A, Baker-Henningham H, Hamadani JD. Effects of a parenting and unconditional cash transfer intervention on child development and maternal well-being in urban Bangladesh: medium-term follow-up of a cluster randomised controlled trial. J Glob Health. 2026;16:04321.

Table S1. Baseline characteristics of participants followed up and lost to follow-up at five years

**Characteristics**	**Followed up[1] N = 555**	**Lost to follow-up[1] N = 44**	**p-value[2]**
Child Age in months	11·3 (3·2)	12·0 (3·1)	0·14
Sex of child, Female	279 (50·3%)	24 (54·5%)	0·60
Mother BMI	22·9 (4·2)	23·2 (4·3)	0·60
Age of Mother in years	25·7 (5·1)	24·8 (5·3)	0·30
Mother's education in years	7·0 (3·5)	7·6 (4·0)	0·30
Father's education in years	5·5 (4·3)	6·7 (4·4)	0·11
Wealth Index			
Poor	186 (33·5%)	15 (34·1%)	0·80
Middle	183 (33·0%)	16 (36·4%)	
Rich	186 (33·5%)	13 (29·5%)	
**Child characteristics**			
Cognitive development	93·9 (10·8)	94·8 (12·9)	0·70
Language development	91·4 (12·6)	91·7 (15·5)	0·90
Motor development	93·8 (14·7)	93·3 (18·0)	0·90
Length-for-age z score (LAZ)	–1·1 (1·1)	–1·3 (1·1)	0·30
Weight for age z score (WAZ)	– 0·7 (1·1)	–0·8 (0·8)	0·14
Weight for Length z score (WLZ)	– 0·1 (1·2)	–0·2 (0·8)	0·30
**Parenting characteristics**			
FCI Total	5·7 (3·3)	5·5 (2·6)	0·70
Father Engagement	0·4 (0·8)	0·3 (0·6)	0·20
**Maternal characteristics**			
Depression	8·7 (4·8)	8·9 (4·3)	0·70
Exposure to violence	265 (47·7%)	24 (54·5%)	0·40
Quality of life			
• *Physical*	65·3 (14·0)	65·4 (13·1)	>0·90
• *Psychological*	60·6 (12·6)	61·8 (11·4)	0·50
• *Social relationship*	58·2 (14·8)	58·3 (17·4)	>0·90
• *Environmental*	49·8 (11·4)	50·8 (9·0)	0·50

[1]Mean (SD); n (%)
[2]Welch Two Sample t-test; Pearson's Chi-squared test; Fisher's exact test as fitted. BMI – body mass index, FCI – family care indicators.

Table S2. Characteristics of participants in the pure control and cash only groups at five-year follow-up

Characteristics	Pure control* N=267	Cash only* N=288	p-value¶
Child age in years	6·9 (0·6)	6·9 (0·3)	0·70
Mother's education in years	4·9 (2·2)	7·2 (3·8)	<0·001
Father's education in years	3·9 (3·3)	6·0 (4·5)	<0·001
Wealth Index			<0·001
Poor	152 (56·9%)	39 (13·5%)	
Middle	90 (33·7%)	80 (27·8%)	
Rich	25 (9·4%)	169 (58·7%)	
Child Height for Age z-score	–0·9 (1·2)	–0·9 (1·0)	0·60
Child Weight for Age z-score	–1·2 (1·1)	–1·1 (1·2)	0·12
Child BMI for Age z-score	–1·0 (1·1)	–0·8 (1·2)	0·09
**Child outcomes**			
Early Grade Mathematics Assessment	38·2 (22·8)	40·9 (20·7)	0·15
Early Grade Reading Assessment	16·7 (14·0)	18·1 (13·0)	0·20
Full Scale IQ	63·4 (11·1)	65·6 (11·2)	0·02
**Parenting outcome**			
Home Total	21·2 (6·0)	27·2 (6·2)	<0·001
**Maternal outcomes**			
Total empowerment	16·7 (4·2)	20·2 (4·5)	<0·001
Depression	11·2 (5·1)	10·9 (4·9)	0·60
Exposure to violence	90 (33·7%)	91 (31·5%)	0·60
Quality of life			
• *Physical*	63·7 (12·5)	61·9 (13·2)	0·10
• *Psychological*	57·9 (13·1)	61·2 (12·8)	0·003
• *Social relationship*	61·0 (16·5)	64·1 (14·8)	0·03
• *Environmental*	47·4 (13·4)	50·5 (12·9)	0·01

*Mean (SD); n (%). ¶Welch Two Sample t-test; Fisher's exact test. BMI – body mass index.

Table S3. Effect of unconditional cash transfer only compared with the pure control group at five-year follow-up

Outcomes	β (95% CI) *	Adjusted ES (95% CI)	ICC	p-value
**Child outcome**				
Full Scale IQ	–0·04 (–2·01, 1·93)	–0·00 (–0·18, 0·17)	0·02	0·97
Early Grade Reading Assessment	–0·45 (–2·78, 1·89)	–0·03 (–0·20, 0·13)	0·03	0·71
Early Grade Mathematics Assessment	–0·81 (–4·40, 2·77)	–0·04 (–0·19, 0·12)	0·03	0·66
**Parenting outcome**				
Home Total	4·72 (3·59, 5·84)	0·78 (0·59, 0·97)	0·11	<0·001
**Maternal outcomes**				
Total empowerment	2·78 (1·97, 3·60)	0·66 (0·47, 0·85)	0·05	<0·001
Depression	0·16 (–0·71, 1·04)	0·03 (–0·14, 0·20)	0·00	0·72
Exposure to violence (Odds Ratio)	0·90 (0·60, 1·35)		0·01	0·61
Quality of life				
• *Physical*	–2·58 (–4·89, –0·27)	–0·21 (–0·39, –0·02)	0·00	0·03
• *Psychological*	1·73 (–0·66, 4·12)	0·13 (–0·05, 0·32)	0·05	0·16
• *Social*	2·12 (–0·65, 4·90)	0·13 (–0·04, 0·30)	0·00	0·13
• *Environmental*	1·68 (–0·45, 3·81)	0·13 (–0·03, 0·28)	0·01	0·12

* Mixed-effects linear regression models were used for continuous variables, and generalized linear mixed models with logit link were used for binary outcomes· All models were adjusted for child age, child sex, mother education, father education, wealth index and tester/interviewer·
CI – confidence interval, ES – effect size, ICC – intracluster correlation coefficient, OR – odds ratio, UCT – unconditional cash transfer.
